# Compliance of checking HbA1c in a tertiary care hospital of Pakistan

**DOI:** 10.12669/pjms.37.1.2814

**Published:** 2021

**Authors:** Abdul Aziz, Syed Ahsan Ali

**Affiliations:** 1 Dr. Abdul Aziz, FCPS (Medicine). Fellow Endocrinology, Diabetes and Metabolism, Department of Medicine, Faculty Office Building, The Aga Khan University Hospital, Karachi, Pakistan; 2 Dr. Syed Ahsan Ali, FCPS (Medicine). Assistant Professor, Department of Medicine, Faculty Office Building, The Aga Khan University Hospital, Karachi, Pakistan

**Keywords:** HbA1c, Compliance, Developing country, Diabetes Mellitus

## Abstract

**Background and Objectives:**

The prevalence of diabetes mellitus worldwide was 171 million one and half decade ago, while the prediction is 366 million patients by 2030 and more than 640 million people by 2040. HbA1c value represents average blood glucose over the past 2-3 months and accounts for both pre-prandial and post-prandial blood glucose levels. A link between HbA1c and diabetic complications has been confirmed. In general, patients with controlled diabetes mellitus should have at least biannual testing, while patients with uncontrolled diabetes mellitus or unmet glycemic targets should be tested every three months. The objective was to see compliance of checking HbA1c in tertiary care hospital of a developing world.

**Methods:**

This was a retrospective observational study done from 1^st^ February 2019 to 31^st^ March 2019 in the Department of Medicine and Surgery, The Aga Khan University Hospital, Karachi. All patients of age 18 years and above, admitted with a diagnosis of diabetes mellitus (DM)from 1^st^ February 2019 to 31^st^ March 2019 were included. If HbA1c was less than 7% the patients were labelled as having controlled DM, otherwise, uncontrolled DM. If HbA1c of patients with controlled DM was not checked in last six months and if HbA1c of patients with uncontrolled DM was not checked in last three months then it was labelled as non-compliance of checking HbA1c.

**Results:**

Out of 1732 diabetic patients only 94 patients fulfilled inclusion criteria. Out of these 94 patients 43 (45.7%) were male. Mean HbA1c was 7.90% (1.4) and 69 (73.4%) patients had uncontrolled diabetes mellitus. Overall, the compliance of checking HbA1c was 58.5%. In uncontrolled diabetes mellitus patients, the compliance of checking HbA1c was 45% and in controlled diabetes mellitus patients the compliance was 96%.

**Conclusion:**

The compliance of checking HbA1c is inadequate in diabetic inpatients. The considerable prevalence of diabetes and the benefits of timely interventions in diagnosed patients to prevent complications suggest the need for a comprehensive awareness among the doctors for checking HbA1c.

## INTRODUCTION

The prevalence of diabetes mellitus worldwide was 171 million one and half decade ago, while the prediction is 366 million patients by 2030.^1^Recent estimates point to more than 640 million people being affected by this disease by 2040.^2^ With clear association with multiple comorbidities such as cardiovascular disease,^3^,^4^ renal disease, infections, Erase malignancy and functional impairment, diabetes places a huge financial burden on both patients and health care systems. In 2012 the American Diabetes Association (ADA) estimated total economic cost of diabetes care in the United States of about $245 billion.^5^

HbA1c value represents average blood glucose over the past 2-3 months and accounts for both pre-prandial and post-prandial blood glucose levels. Regular HbA1c measurement is recommended for all patients with diabetes mellitus for the assessment of blood glucose control.^1^,^6^,^7^ In diabetes care, HbA1c measurement has been considered one of the most important laboratory advances. A link between HbA1c and diabetic complications has been confirmed and the need for adequate blood glucose control underscored.^8^ However, compliance to these recommendations is very low.^9^ Measurement of HbA1c was infrequent, occurring in only 18.4% of encounters where diabetes mellitus was included as an admission diagnosis in a study by Beatastrack et al.^10^ In general, patients with controlled diabetes mellitus should have at least biannual testing, while patients with uncontrolled diabetes mellitus or unmet glycemic targets should be tested every three months.^6^

On the robust literature search there is little data available on the compliance of checking HbA1c in developing countries. No data is available from our country. By checking HbA1c timely, we can modify the treatment and reduce complications of diabetes mellitus. This provides strong rationale to assess the magnitude of compliance of checking HbA1c.

## METHODS

This was a retrospective observational study from 1^st^ February 2019 to 31^st^ March 2019 conducted in the Department of Medicine and Surgery, The Aga Khan University Hospital, Karachi. The study was approved by Ethical review committee of The Aga Khan University Hospital (ERC No # 2019-1464-3628). Non probability, consecutive sampling was adopted. All patients of age 18 years and above, of both genders, admitted in hospital with a diagnosis of diabetes mellitus were included. All patients who had HbA1c of less than 7% were said to have controlled diabetes mellitus and those who had HbA1c equal to or more than 7% were said to have uncontrolled diabetes mellitus. A patient was said to be compliant if HbA1c was checked at or within three months for uncontrolled diabetes mellitus and at or within six months for controlled diabetes mellitus. Those patients who were diagnosed as diabetes mellitus in the latest admission and those whose data of HbA1c is not available for last six months for controlled diabetes mellitus and last three months for uncontrolled diabetes mellitus were excluded. Analysis was done by SPSS software package. To determine the association between the variables *P* <.05 was supposed to be significant.

## RESULTS

A total of 1732 diabetic patients were admitted from 1^st^ February 2019 to 31^st^ March 2019 in The Aga Khan University Hospital Karachi, out of which only 94 patients fulfilled our inclusion criteria and were selected in our study. Out of 94 diabetic patients 43 (45.7%) were male. The mean age was 62.6 (14.1) years and mean HbA1c was 7.90% (1.4). 69 (73.4%) patients had uncontrolled diabetes mellitus while 25 (26.6%) had controlled diabetes mellitus. Overall, the compliance of checking HbA1c was 58.5%. Within uncontrolled diabetes mellitus the compliance of checking HbA1c was found in 31(45%) and within controlled diabetes mellitus the compliance was found in 24 (96%) (*P* 0.00). The baseline characteristics of study subjects are given in Table-I and overall compliance in controlled and uncontrolled diabetes mellitus is given in Table-II.

**Table-I:** Characteristics and compliance of checking HbA1c.

		Value (SD)
Age (years)		62.6 (14.1)
Gender	Male	43 (45.7%)
	Female	51 (54.3%)
HbA1c (%)		7.90 % (1.4)
Uncontrolled Diabetes Mellitus		69 (73.4%)
Controlled Diabetes Mellitus		25 (26.60%)
Compliant		55 (58.5%)
Non-compliant		39 (41.5%)

**Table-II:** Compliance of checking HbA1c in controlled and uncontrolled diabetes mellitus.

		*P* value
Overall (Total 94)	55 (58.5%)	
In patients with controlled diabetes mellitus (Total 25)	24 (96%)	0.00
In patients with uncontrolled diabetes mellitus (Total 69)	31 (45%)	

The overall compliance in medicine was 49 (55.6%) and in surgery it was 6 (37.5%). In medicine subspecialties the compliance in the descending order of frequency was neurology eight (88.9%), pulmonology 8 (80%), cardiology nine (69.2%), internal medicine 23 (60.5%), gastroenterology 1 (33.3%) and nephrology 0(0%).

## DISCUSSION

In our study the compliance rate of checking HbA1c was inadequate. The compliance rate was even poorer in those patients with uncontrolled diabetes mellitus as compared to controlled diabetes mellitus. The overall compliance rate was 58.5% in our study which is similar to the study by Graham Woodward et al, in which the compliance of checking HbA1c was 58%.^11^ Our results are also similar to those of a smaller Canadian study of physician charts, which found that only 53% of patients with Type-2 diabetes had HbA1c testing during one year.^12^ However, these studies were carried out in developed countries and in outpatient settings.

In a study conducted in primary healthcare in Saudi Arabia by Siddqui MS et al the compliance was 39%.^13^ In a study done in Bahrain HbA1c was done twice a year in 20% of patients.^14^An Ethiopian study found that none of study subjects had HbA1c determination.^15^ A study by Biatastrack et al showed that measurement of HbA1c was infrequent, occurring in only 18.4% of encounters where diabetes mellitus was included as an admission diagnosis which is lower as compared to our study.^10^

In our study the compliance was poorer in uncontrolled diabetes mellitus (45%) as compared to controlled diabetes mellitus (96%). This finding is interesting as physicians and surgeons should be more vigilant in assessing status of diabetic patients with uncontrolled diabetes mellitus. However, in our study physicians and surgeons were less vigilant for patients with uncontrolled diabetes mellitus. The reason for this is unknown. It may be merely due to by chance.

Sub specialty-wise further analysis of our data showed that compliance of checking HbA1c was higher in the department of medicine as compared to surgery. The compliance was highest in the sub-specialties (e.g. neurology and cardiology) where diabetes mellitus is an important risk factor for the diseases like stroke and myocardial infarction.

To the best of our knowledge there is no inpatient study in South Asia on the compliance of checking HbA1c which is an important predictor of determining the control of diabetes mellitus. Our study was carried out only in inpatient setting and the resultant sample size was relatively small. Large scale studies including outpatient setting patients are required.

### Limitation of the Study:

1. .It was inpatient study and a small number of patients were included.
2. The study subjects were not classified as type 1 diabetes mellitus, type 2 diabetes mellitus and gestational diabetes mellitus.

## CONCLUSIONS

The compliance of checking HbA1c is inadequate in diabetic inpatients. The considerable prevalence of diabetes and the benefits of timely interventions, on the basis of HbA1c values, in diagnosed patients to prevent complications suggest the need for more awareness among the doctors for checking HbA1c.

### Authors’ Contribution:

**AA:** Conceived, designed, did data collection, manuscript writing and responsible for the integrity of the study.

**SAA:** Did statistical analysis, review and final approval of manuscript.
